# Total Kidney Volume, Hypertension, and Deterioration of Kidney Function in Children with Early-Stage ADPKD

**DOI:** 10.3390/jcm14134498

**Published:** 2025-06-25

**Authors:** Agnieszka Turczyn, Grażyna Krzemień, Dominik Nguyen, Katarzyna Smyk

**Affiliations:** 1 Department of Pediatrics and Nephrology, Medical University of Warsaw, 02-091 Warsaw, Poland; grazyna.krzemien@wum.edu.pl (G.K.); smyk_kasia@wp.pl (K.S.); 2 Department of Pediatric Radiology, Medical University of Warsaw, 02-091 Warsaw, Poland; dominik.nguyen@op.pl

**Keywords:** ADPKD, children, total kidney volume (TKV), ABPM, hypertension, hyperfiltration, kidney function

## Abstract

**Background:** Several studies have shown that total kidney volume (TKV) measurements may serve as a non-invasive imaging biomarker for monitoring and predicting the progression of autosomal dominant polycystic kidney disease (ADPKD) in children. **Methods**: This study aimed to evaluate the relationship between height-adjusted TKV (htTKV), estimated glomerular filtration rate (GFR), and blood pressure, assessed using 24 h ambulatory blood pressure monitoring (ABPM), in children with early-stage ADPKD. The study was conducted with 72 children, mean age 12.46 ± 3.76 (5.42–17.92). **Results:** Hypertension (HT) was diagnosed in (20) 28% of children. ABPM allowed the identification of previously undiagnosed HT in 12 (16.7%) children. Decreased GFR was demonstrated in 10 (14%) children, and hyperfiltration in 5 (7%) children. Significantly higher htTKV and calculated TKV z-score and more frequent decreases in GFR were observed in hypertensive children (*p* = 0.018; 0.020 and 0.010, respectively). The study demonstrated a significant inverse correlation between htTKV and GFR (r −0.25; *p* = 0.032). The TKV z-score showed a very good correlation with all ABPM parameters, except for DBP and DBP z-score during the day. Receiver operating curve (ROC) analysis showed that htTKV and TKV z-score had good diagnostic value for predicting a decline in GFR (AUC 0.808, *p* < 0.001), but were not useful for predicting the onset of HT (AUC 0.697, *p* = 0.010). **Conclusions:** There is a relationship between TKV, GFR, and blood pressure parameters in children with early-stage ADPKD. The TKV z-score can be useful for predicting GFR decline. Children with ADPKD and increasing TKV require careful blood pressure monitoring.

## 1. Introduction

Autosomal dominant polycystic kidney disease (ADPKD) is one of the most common hereditary human monogenic diseases, affecting 1:1000 to 4000 individuals in Europe [1]. It is characterized by a continuous development of multiple cysts in the kidneys that replace normal renal parenchyma, cause bilateral kidney enlargement, and ultimately lead to renal failure [2]. Approximately 5% of ADPKD cases manifest during childhood [3] with 3% of children being diagnosed in utero or within the first 18 months of life, which is considered a very early onset of the disease [4].

Chronic kidney disease (CKD) in ADPKD usually develops in middle age, whereas enlargement of the kidney size, hypertension (HT), glomerular hyperfiltration, hematuria, and proteinuria may occur already in childhood [4]. The presence of hypertension in ADPKD children is associated with a decline in kidney function and the progression of cardiovascular disease. Ambulatory blood pressure monitoring (ABPM) is considered an essential tool for the detection and treatment of hypertension. It also allows for identification of patients with masked hypertension, isolated clinic hypertension, and abnormal blood pressure (BP) profiles [5].

Several studies have shown that measurements of total kidney volume (TKV) may serve as a noninvasive imaging biomarker for monitoring and predicting disease progression, as well as a major prognostic end point in clinical trials in patients with ADPKD [6,7,8,9]. The measurement of TKV by ultrasound (US) is the method of choice for assessing kidney dimensions in children due to the high accessibility and cost-effectiveness of US compared to other modalities such as magnetic resonance imaging (MRI) and computed tomography (CT). Only a few studies are evaluating TKV with US, kidney function, and ABPM in children with ADPKD [8,10,11].

The aim of this study was to evaluate the relationship between adjusted TKV measured by US, kidney function, and blood pressure parameters in children with early-stage ADPKD.

## 2. Materials and Methods

### 2.1. Study Group

This cross-sectional single-center study was conducted with 72 children with ADPKD, who were treated from 2021 to 2024 at a the tertiary pediatric nephrology center in Poland. Inclusion criteria were as follows: age < 18 years, height ≥ 120 cm, and ADPKD diagnosis established in patients with at least one kidney cyst and positive family history or proven mutation in PKD1 or PKD2 gene [4,12]. Exclusion criteria were as follows: height < 120 cm due to lack of reference values for interpretation of ABPM, cystic kidney disease other than ADPKD, congenital kidney and urinary track abnormalities, or secondary causes of hypertension. In all participants, the following clinical data were evaluated: age [years], sex, body height [cm], and body weight [kg]. The glomerular filtration rate (GFR mL/min/1.73 m^2^) was estimated according to the revised 2009 Schwartz formula [13]. According to KDIGO guidelines, GFR values between 90–140 mL/min/1.73 m^2^ were considered normal [14,15].

### 2.2. Kidney Ultrasound

Kidney volume (KV) was measured by performing standard ultrasonography. The ellipsoid formula [16] was used to calculate each kidney volume: π/6 × length [cm] × width [cm] × depth [cm]. The TKV z-score (number of standard deviations) of each kidney according to height was calculated by the use of a calculator found at www.pedkidneysize.com. The Lambda, Mu and Sigma (LMS) method was used to calculate the z-score. TKV z-score result of less than 2 corresponding to a TKV less than the 97th percentile and was considered normal [17]. Mean total kidney volume (TKV) was obtained by summing the volume of the left and right kidney, dividing by 2 and adjusting to height (htTKV), expressed in cm^3^/m. The average z-score was calculated similarly to the mean TKV. To compare TKV’s impact on blood pressure parameters and renal function, children were divided into 3 groups according to their TKV z-score: normal renal volume—TKV z-score < 2, moderately increased kidney volume—TKV z-score 2–4, and highly increased kidney volume—TKV z-score > 4.

### 2.3. Blood Pressure

ABPM was performed using the SunTech Oskar 2 device (SunTech Medical Inc., Morrisville, NC, USA) according to American Heart Association guidelines [18]. BP was automatically recorded every 15 min from 7 a.m. to 10 p.m. and every 30 min from 10 p.m. to 7 a.m. The recordings were adequate for clinical interpretation if at least 75% of the measurements were valid. Periods of activity and resting were assessed according to patients’ individual diaries. Systolic, diastolic, and mean arterial pressure (SBP, DBP, and MAP, respectively), blood pressure loads, and nighttime blood pressure dipping (DIP) were measured. Patients were classified as non-dippers if their dipping status was less than 10%. SBP, DBP, and MAP were also expressed as z-scores [19]. BP loads were evaluated as the percentage of BP measurements above the 95th percentile for sex and height. The diagnostic criteria for HT based on European and North American guidelines were mean SBP and/or DBP ≥ 95th percentile or use of antihypertensive medications for the indication of HT [5,18]. Isolated nocturnal HT was diagnosed in the presence of hypertension during nighttime and the presence of normal values of BP during the awake period. High-normal BP was defined as mean SBP and/or DBP ≥ 90th and < 95th percentile, and normal BP as SBP and/or DBP < 90th percentile [5].

### 2.4. Ethical Issues

The study was approved by the local Bioethics Committee for Human Research (No. KB/112/A2021). The clinical investigation was conducted in accordance with the ethical standards of the institutional research committee and the guidelines of the Declaration of Helsinki. Informed consent was obtained from all participant representatives and participants (≥16 years) prior to study inclusion.

### 2.5. Statistical Analysis

Statistical analysis was performed using Statistica 13.3 PL software (StatSoft, Tulsa, OK, USA). The results were presented as the mean ± standard deviation (SD) and minimum and maximum ranges or the median and interquartile ranges (IQR, 25–75), and categorical variables as absolute frequencies and percentages. The normality of variables was analyzed using the Shapiro–Wilk test. Student’s *t*-test or the Mann–Whitney U-test (depending on the variables distribution), and the chi-square and Fisher’s exact test, were used to compare two groups of variables. ANOVA and post hoc tests were performed to analyze differences between the three subgroups. The relationship between variables was evaluated using Pearson’s or Spearman’s rank correlation. Receiver operating curve (ROC) analysis was used to calculate the area under the curves (AUC) for kidney volume and to find the best cut-off values (including 95% CI), sensitivity, specificity in the detection of GFR decrease, and presence of HT for each variable. *p* values less than 0.05 were considered statistically significant for all tests.

## 3. Results

Seventy-two children with ADPKD were recruited for the study (Table 1). A positive family history of ADPKD was found in 88% of children. In cases of negative family history, genetic tests revealed a mutation in the PKD1 gene. No PKD2 mutation was found. Estimated GFR values were normal in 79% of children, a decrease in GFR values was observed in 14% of children, and hyperfiltration was observed in 7% of children. Twenty (28%) children met the criteria for HT. Diagnosis of HT was established in children between 2 and 16 years. In two girls, HT was detected at the age of 2 and 3 years. These girls are sisters and abnormal renal echogenicity was observed already at fetal age. Eight patients had been diagnosed with HT before being included in the study. They were under the treatment of HT at the time of ABPM recording. Four of these children had unsatisfactory blood pressure control at the moment of performing ABPM and required modification of therapy. Twelve children (16.7%) were for the first time diagnosed with HT at the initial examination by using ABPM. All of them had normal BP values during ambulatory oscillometric measurements. Sixteen children with hypertension received angiotensin-converting enzyme inhibitors (ACEIs) in monotherapy. One child received a beta-blocker due to a high heart rate. Three children were taking two antihypertensive medications: ACEIs and calcium channel inhibitors.

Comparisons of demographic, ultrasonography, kidney function, and ABPM data in children with normal BP and hypertension are presented in Table 2. Hypertensive children were characterized by significantly higher values of kidney volume and lower GFR values. A decrease in GFR was observed in 30% of hypertensive children, significantly more often than in normotensive children. Patients with HT had significantly higher SBP, DBP, and MAP during a 24 h period, day, and night, compared to normotensive children (all *p* < 0.001). SBP 24 h loads and DBP 24 h loads were significantly higher in hypertensive children than in those with normal BP. SBP non-dippers represented 21 (29%) of 72 children, DBP non-dippers 8 (11%), and extreme SBP or DBP dipping 26 (36.1%) children. The percentage of children with no drop in nocturnal blood pressure >10%, as well as the percentage of children who were extreme dippers, was comparable in both groups.

Comparison of age, GFR, and ABPM data with kidney volume are reported in Table 3. There were no significant differences in GFR values between TKV z-score groups. None of the children with a TKV z-score < 2 had a GFR decrease of less than 90 mL/min/1.73 m^2^. The percentage of children with reduced GFR increased with higher TKV z-scores: 8% for z-scores between 2 and 4, and 36% for z-scores greater than 4. Hyperfiltration was present in all patient groups. Hypertension was diagnosed more often in patients with TKV z-score >4 (55%), and less often in those with TKV z-score 2–4 (25%) and TKV z-score <2 (20%).

**Figure 1:**
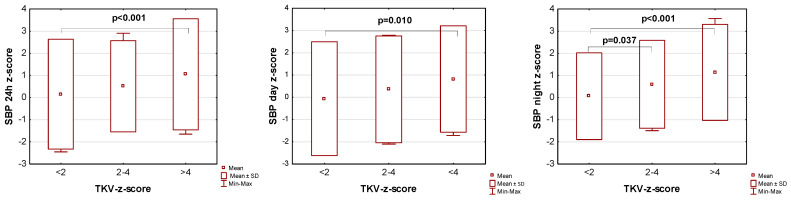
Influence of kidney volume on mean systolic blood pressure during 24 h, day, and night time periods. SBP: systolic blood pressure, TKV: total kidney volume.

**Figure 2:**
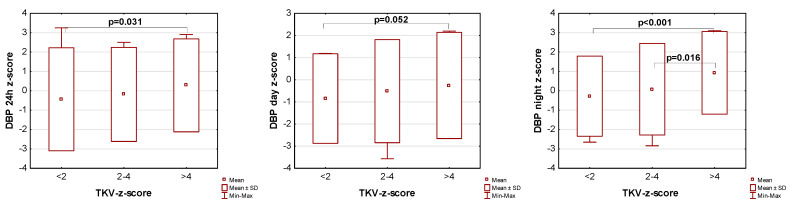
Influence of kidney volume on mean diastolic blood pressure during 24 h, day, and night time periods. DBP: diastolic blood pressure, TKV: total kidney volume.

**Figure 3:**
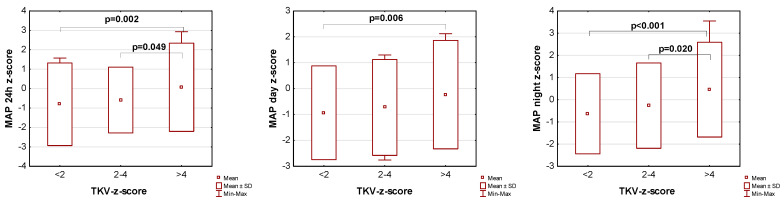
Influence of kidney volume on mean arterial pressure during 24 h, day, and night time periods. MAP: mean arterial pressure, TKV: total kidney volume.

The influence of kidney volume on blood pressure in ADPKD children is presented in Figure 1, Figure 2 and Figure 3. Children with TKV z-score >4 had significantly higher mean SBP z-score and MAP z-score for 24 h, day, and night time periods, as well as significantly higher mean DBP z-score for 24 h and night periods compared to those with TKV z-score <2. The difference between these groups regarding SBP z-score during the day almost reached statistical significance (*p* = 0.053). Patients with TKV z-score >4 also demonstrated significantly higher mean DBP z-score during the night, mean MAP z-score over 24 h, and MAP z-score during the night compared to those with TKV z-score 2–4. Patients with TKV z-score 2–4 demonstrated significantly higher mean SBP z-score during the night than patients with TKV z-score <2.

The study demonstrated a mutual correlation between kidney volume, GFR, and BP values (Table 4). There were significant positive correlations of htTKV and TKV z-score with age, and a significant inverse correlation between htTKV and GFR. TKV z-score showed very good correlation with all ABPM parameters, except DBP and DBP z-score during the day. htTKV demonstrated slightly worse or no correlation with SBP z-score, DBP z-score, and MAP z-score during 24 h, day, and night time periods, and very good correlation with other parameters. Kidney volume parameters significantly positively correlated with SBP loads and DBP loads.

The study also presents a significant positive correlation of GFR with DBP, DBP z-score, and MAP during the day, as well as with MAP and MAP z-score during 24 h in hypertensive children (Table 5).

**Figure 4:**
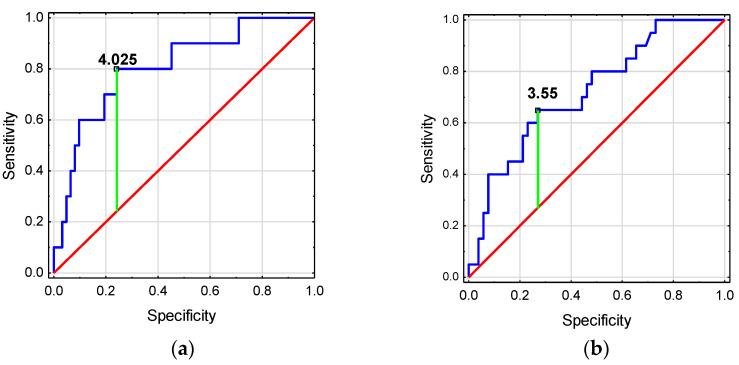
Diagnostic usefulness of kidney volume for predicting (**a**) the occurrence of GFR decline in children with ADPKD, (**b**) the occurrence of the onset of hypertension in children with ADPKD.

The ROC analysis showed that htTKV and TKV z-score have good diagnostic values for detecting deterioration in GFR (Figure 4a). TKV z-score ≥ 4.025 was associated with a higher risk of GFR decline <90 mL/min/1.73 m^2^. Meanwhile, htTKV and TKV z-score have low diagnostic value for the detection of HT in ADPKD children (Figure 4b, Table 6).

## 4. Discussion

Our study is one of a very small number of studies evaluating children in the early stages of ADPKD. We confirmed that enlarged kidney volume, impaired kidney function presented as hyperfiltration or decreased GFR, as well as unidentified hypertension, may be present in children in the early stage of ADPKD. We were one of the few who showed an association between renal TKV and GFR and blood pressure parameters in children with ADPKD. For the first time, we investigated which TKV z-score increased the risk of reduced GFR and the presence of hypertension in children with ADPKD.

Hypertension is one of the most important risk factors for the fast progression of CKD. According to a meta-analysis performed by Marlais, in the pediatric ADPKD population, the presence of HT is estimated at 20% [20]. Based on the analysis of ABPM data, the prevalence of HT is even higher and is estimated at 35% [12]. The incidence of HT increases significantly over time in children and young adults with ADPKD [20]. Seeman et al. revealed a 2 percentage points per year increase in the prevalence of HT in children with ADPKD during 6 years of follow-up [21]. Data were analyzed according to blood pressure values collected using a standard method. Unfortunately, they did not perform ABPM in all patients. The randomized clinical trial performed by Cadnapaphornchai et al. showed that half of ADPKD children with BP in the range of 75–90th percentile and almost one-third of patients with a BP range below 75th percentile developed HT within 5 years of the study [10]. What is more, children with borderline hypertension (75–95th percentile) demonstrated a decline in kidney function over time and have increased LVMI compared to children with BP lower than the 75th percentile [22]. According to our ABPM data, 28% of children were hypertensive, 14% of children had BP in the 75–90th percentile range, and 7% had BP in the 90–95th percentile range.

Notably, there is a risk that some hypertensive children with ADPKD are not being identified because of the normal BP measurements during medical appointments. ABPM seems to be a more precise method for detection of HT compared to office blood pressure measurements (34% vs. 16%) [23]. In an observational prospective study, Sans Atxer et al. demonstrated that almost 30% of examined children with ADPKD had not been diagnosed with HT without ABPM due to masked HT [11]. Also, Seeman et al. showed that nearly 60% of patients with daytime HT according to ABPM had normal BP values during regular measurements [24]. We first diagnosed HT in 16.7% of children by using ABPM at the initial examination. All of them had normal BP values during earlier ambulatory oscillometric measurements.

In the large prospective study, which included early-stage ADPKD children, Massella et al. found higher SBP and DBP during the night compared to the day. A total of 18% of ADPKD children had been diagnosed with isolated nocturnal HT, and 52% of children were non-dippers [12]. Chapman et al. demonstrated that more than 25% of children showed a lack of nocturnal decline in blood pressure [25]. Marlais showed a lack of nocturnal dipping in 35% of children [26]. We had 29% SBP non-dippers and 11% DBP non-dippers. To date, studies from the adult population have described a significant association between nocturnal HT, lack of nocturnal reduction in BP, and organ damage, especially a significantly higher risk of adverse cardiovascular events [27].

US, due to its wide availability, may be the preferred method for calculating KV in children with ADPKD. The Bland–Altman chart showed no significant differences between TKV measurements by US and MRI techniques in children with ADPKD [28]. Although the US can be less accurate in assessing kidney volume when kidney size exceeds 17 cm, it is suggested to be an appropriate tool for determining the severity of the disease and the patient’s prognosis in ADPKD [29,30]. An increase in TKV showed exponential-like growth from children to adulthood, individually for each patient with ADPKD [10,31]. Initial TKV may influence the rate of TKV increase and GFR decrease. High baseline TKV may result in a more rapid increase in TKV and a decrease in GFR [32]. Few studies showed that ADPKD children, in particular those with HT, had a significantly greater increase in TKV calculated by the US and MRI methods [7,33,34,35]. The Consortium for Radiologic Imaging Studies of Polycystic Kidney Disease (CRISP) study also reported the relationship between increased TKV and the presence of HT in young ADPKD patients with normal renal function [36]. Consistent with those studies, we also observed that children with HT had greater TKV and significantly lower GFR than normotensive ones. Children with TKV z-score >4 had significantly higher SBP and MAP during 24 h, day, and night time periods, and significantly higher DBP during 24 h and night than children without kidney enlargement. In accordance with the study performed by Gurusinghe et al., who showed a significant association between higher systolic BP values in 24 h, day, and night time periods, systolic BP loads, and TKV [8], we also found a significant correlation between mean htTKV, mean TKV z-score, and blood pressure parameters.

Most children with ADPKD have renal function within the normal range. According to the meta-analysis performed by Marlais, the prevalence of reduced renal function defined as GFR < 90 mL/min/1.73 m^2^ in ADPKD children is 8% (95% CI 2% to 26%) [20]. A significant decrease in GFR was also observed by Cadnapaphornchai et al. during 5 years of follow-up in pediatric ADPKD patients with mild disease [10]. In a study performed by Seeman et al., the percentage of ADPKD children with stage 2 CKD doubled at the final visit from 5 to 10% [21]. Similarly, we detected a decline in renal function in 14% of our patients. In a meta-analysis performed by Woo Ri Jo et al., the authors reported a significant negative correlation between TKV, TKV growth, and GFR or GFR decline rates in adults [37]. The CRISP study documented an inverse correlation between renal volume and GFR in patients with ADPKD under 25 years of age [36]. We were one of the few who confirmed a negative correlation between htTKV measured in US and GFR in children with the early stage of ADPKD.

Data highlight the correlation between the presence of hyperfiltration and the severity of ADPKD [10,32]. Seeman showed that 19% of ADPKD children had hyperfiltration at the initial visit and 14% at the final visit. In children with initial hyperfiltration, the GFR significantly decreases during the follow-up [21]. Similarly, Helal et al. observed that children with hyperfiltration at baseline experienced a faster decline in GFR in the following 5 years [15]. In our cohort, 7% of children had hyperfiltration. The presence of hyperfiltration was similar regardless of kidney enlargement and values of blood pressure. The presence of hyperfiltration may be the reason why the lack of correlation between TKV z-score and GFR occurred. We can speculate that the presence of a significant positive correlation between GFR and DBP during the day and MAP during 24 h and the day in hypertensive children might result from the occurrence of hyperfiltration.

In adults, the development of stage III CKD can be predicted by an ultrasound measurement of htTKV >650 mL/m^2^ [29]. Our study revealed a good prognostic value of htTKV and TKV z-score for assessing decline in GFR <90 mL/min/1.73 m^2^. None of the children with a normal TKV z-score had a decreased GFR. As the TKV z-score increased to 2–4 and then to over 4, the proportion of children with a reduced GFR increased, reaching 8% and 36%, respectively. We were the first to indicate an increased risk of reduced GFR <90 mL/min/1.73 m^2^ from a TKV z-score >4 in ADPKD children. htTKV and TKV z-score had low usefulness in predicting the occurrence of HT in children with ADPKD.

Our study has some limitations. The cross-sectional nature of the study makes it impossible to draw causal conclusions. Single-center trial design and a relatively small number of patients may introduce a bias. A further limitation is the lack of repeated evaluation of our patients, which could provide more information about the prevalence of hypertension and impairment of kidney function in our study group.

## 5. Conclusions

There is a relationship between TKV, GFR, and blood pressure parameters in children with the early stage of ADPKD. US measurement of TKV can be helpful in assessing the severity of the disease. In particular, htTKV and TKV z-score can be useful for predicting decline in renal function in children with ADPKD. Children with ADPKD and increasing TKV require careful blood pressure monitoring. Use of ABPM allows early diagnosis of HT in children with ADPKD and therefore early initiation of treatment.

## Figures and Tables

**Table 1:** Demographic, kidney function, and blood pressure data.

Variables	Study Group *n* = 72
Male, *n* [%]	33 (46)
Age at ADPKD diagnosis [years]	5.04 (0.71; 9.71)
Positive family history, *n* [%]	63 (88)
Genetic diagnosis, *n* [%]	9 (12)
Age at examination [years]	12.46 ± 3.75 (5.42–17.92)
Kidney function	
GFR [mL/min/1.73 m^2^]	109.87 ± 18.82 (71.55–155.91)
Normal GFR, [%]	57 (79)
GFR < 90 mL/min/1.73 m^2^, *n* [%]	10 (14)
GFR > 140 mL/min/1.73 m^2^, *n* [%]	5 (7)
Blood pressure	
Normal BP, *n* [%]	52 (72)
BP < 75 pc	37 (71)
BP 75–90 pc	10 (19)
BP 90–95 pc	5 (10)
Age at HT diagnosis	10.27 ± 3.70 (2.33–16.42)
Hypertension, *n* [%]	20 (28)
Systolic and diastolic	14 (70)
Isolated systolic	5 (25)
Isolated nocturnal	1 (5)

ADPKD: autosomal dominant polycystic kidney disease; GFR: estimated glomerular filtration rate; HT: hypertension; BP: blood pressure; pc: percentile.

**Table 2:** Comparison of demographic, ultrasonography, kidney function, and ABPM data in children with normal BP and hypertension.

Variables	Normal BP *n* = 52	Hypertension *n* = 20	*p*
Male, *n* [%]	23 (43)	10 (50)	0.803
Age at ADPKD diagnosis [years]	5.75 (0.67; 12.42)	4.46 (0.75; 6.75)	0.151
Age at examination [years]	11.94 ± 3.73 (5.42–18.00)	12.79 ± 3.27 (8.00–17.92)	0.165
**Ultrasonography**			
TKV [cm^3^]	142.60 (93.45; 198.98)	199.26 (130.15; 267.50)	0.013
htTKV [cm^3^/m]	91.60 (71.53; 115.48)	127.53 (80.82; 158.38)	0.018
TKV z-score	2.52 (1.35; 3.47)	4.59 (2.49; 5.62)	0.020
**Kidney function**			
GFR [mL/min/1.73 m^2^]	112.80 ± 17.15 (75.81–155.91)	101.07 ± 21.28 (71.56–146.62)	0.021
GFR < 90 mL/min/1.73 m^2^, *n* [%]	4 (8)	6 (30)	0.010
GFR > 140 mL/min/1.73 m^2^, *n* [%]	3 (6)	2 (10)	0.314
**ABPM**			
SBP non-dipper, *n* [%]	16 (31)	5 (25)	0.703
DBP non-dipper, *n* [%]	5 (10)	3 (15)	0.363
SBP/DBP > 20% dipper, *n* [%]	18 (35)	8 (40)	0.562
SBP 24 h loads *	6 (3; 15)	53 (23; 65)	<0.001
DBP 24 h loads *	4 (3; 10)	24 (9.5; 30.5)	<0.001

ABPM: 24-h automatic blood pressure measurement; BP: blood pressure; TKV: total kidney volume; htTKV: height-adjusted total kidney volume; GFR: estimated glomerular filtration rate; SBP: systolic blood pressure; DBP: diastolic blood pressure; * SBP and DBP loads were reported as median and IQ ranges.

**Table 3:** Comparison of demographic, kidney function, and ABPM data with kidney volume.

Variables	TKV			*p*		
	z-Score < 2	z-Score 2–4	z-Score > 4	1 vs. 2	1 vs. 3	2 vs. 3
Patients, *n* [%]	26 (36.1)	24 (33.3)	22 (30.6)	-	-	-
Age [years]	11.61 ± 3.72 (6.50–17.83)	12.64 ± 3.87 (5.62–17.92)	13.25 ± 3.63 (6.08–17.92)	0.595	0.289	0.845
Kidney function						
GFR [mL/min/1.73 m^2^]	110.08 ± 14.62 (90.63–146.62)	114.63 ± 18.57 (78.43–155.91)	104.44 ± 22.56 (71.55–155.45)	0.393	0.300	0.069
Normal GFR, *n* [%]	25 (96)	20 (84)	12 (55)	-	-	-
↓ GFR, *n* [%]	0	2 (8)	8 (36)	-	-	-
↑ GFR, *n* [%]	1 (4)	2 (8)	2 (9)	-	-	-
ABPM						
Normal BP	22 (85)	19 (79)	11 (50)	-	-	-
Hypertension, *n* [%]	4 (15)	5 (21)	11 (50)	-	-	-
SBP 24 h loads *	5 (2; 19)	9 (4; 20)	28.5 (10; 59)	1.0	<0.001	0.021
DBP 24 h loads *	5 (3; 10)	4 (3; 15)	10.5 (8; 18)	1.0	0.027	0.063
SBP non-dipper, *n* [%]	5 (19)	8 (33)	8 (36)	0.374	0.193	0.647
DBP non-dipper, *n* [%]	3 (12)	3 (12.5)	2 (9)	0.288	0.562	0.543
SBP/DBP > 20% dipper, *n* [%]	11 (42)	11 (46)	4 (18)	0.898	0.056	0.046

GFR: estimated glomerular filtration rate; TKV: total kidney volume; ABPM: 24 h automatic blood pressure measurement; SBP: systolic blood pressure; DBP: diastolic blood pressure; MAP: mean arterial pressure; * SBP and DBP loads were reported as median and IQ ranges. ↓ GFR: <90 mL/min/1.73 m^2^, ↑ GFR: >140 mL/min/1.73 m^2^.

**Table 4:** Correlation between kidney volume, kidney function, and blood pressure.

Variable	htTKV		TKV z-Score	
	*r*	*p*	*r*	*p*
Age	0.44	<0.001	0.24	0.041
GFR	−0.25	0.032	−0.17	0.154
				
SBP 24 h	0.44	<0.001	0.41	<0.001
SBP 24 h z-score	0.21	0.076	0.28	0.017
SBP day	0.39	0.001	0.36	0.002
SBP day z-score	0.20	0.093	0.27	0.020
SBP night	0.47	<0.001	0.44	<0.001
SBP night z-score	0.30	0.010	0.37	0.001
				
DBP 24 h	0.30	0.010	0.27	0.020
DBP 24 h z-score	0.25	0.037	0.24	0.039
DBP day	0.23	0.050	0.22	0.065
DBP day z-score	0.19	0.116	0.19	0.108
DBP night	0.39	0.001	0.41	<0.001
DBP night z-score	0.38	0.001	0.41	<0.001
				
MAP 24 h	0.39	0.001	0.38	0.001
MAP 24 h z-score	0.26	0.027	0.32	0.006
MAP day	0.33	0.004	0.33	0.005
MAP day z-score	0.23	0.053	0.28	0.018
MAP night	0.47	<0.001	0.46	<0.001
MAP night z-score	0.32	0.006	0.38	0.001
				
SBP loads	0.38	0.001	0.30	0.012
DBP loads	0.36	0.002	0.36	<0.001

htTKV: height-adjusted total kidney volume; GFR: estimated glomerular filtration rate; SBP: systolic blood pressure; DBP: diastolic blood pressure; MAP: mean arterial pressure.

**Table 5:** Correlation between GFR and blood pressure parameters in children with hypertension.

Variable	GFR	
	*r*	*p*
SBP 24 h	−0.09	0.715
SBP 24 h z-score	0.15	0.535
SBP day	−0.01	0.982
SBP day z-score	0.24	0.310
SBP night	−0.20	0.396
SBP night z-score	0.05	0.845
		
DBP 24 h	0.40	0.083
DBP 24 h z-score	0.43	0.061
DBP day	0.49	0.028
DBP day z-score	0.50	0.024
DBP night	0.07	0.767
DBP night z-score	0.06	0.796
		
MAP 24 h	0.52	0.023
MAP 24 h z-score	0.52	0.023
MAP day	0.45	0.053
MAP day z-score	0.51	0.024
MAP night	0.11	0.651
MAP night z-score	0.17	0.482
		
SBP loads	−0.19	0.445
DBP loads	0.45	0.049

htTKV: height-adjusted total kidney volume; GFR: estimated glomerular filtration rate; SBP: systolic blood pressure; DBP: diastolic blood pressure; MAP: mean arterial pressure.

**Table 6:** Diagnostic usefulness of kidney volume for predicting (a) the occurrence of GFR decline in children with ADPKD, (b) the occurrence of the onset of hypertension in children with ADPKD.

Variable	AUC	*p*	Cut-Off	Sensitivity (%)	Specificity (%)	Accuracy (%)
(a)						
htTKV [cm^3^/m]	0.815 (0.692–0.937)	<0.001	128.7	80	82	82
TKV z-score	0.808 (0.663–0.953)	<0.001	4.025	80	76	76
(b)						
htTKV [cm^3^/m]	0.687 (0.539–0.836)	0.014	149.31	44	93	81
TKV z-score	0.697 (0.547–0.846)	0.010	3.55	61	76	72

TKV: total kidney volume; htTKV: height-adjusted total kidney volume; GFR: estimated glomerular filtration rate.

## Data Availability

The data analyzed in this study are available from the corresponding author on reasonable request.
